# Shaping undergraduate public health education through critical race theory: a case study

**DOI:** 10.3389/fpubh.2023.1192771

**Published:** 2023-08-25

**Authors:** Michelle A. Tagorda-Kama, Uday Patil, Jane J. Chung-Do, Lisa Kehl, Mapuana C. K. Antonio, Denise C. Nelson-Hurwitz

**Affiliations:** Office of Public Health Studies, Thompson School of Social Work and Public Health, University of Hawai‘i at Mānoa, Honolulu, HI, United States

**Keywords:** public health education, health professionals, critical race theory, racism, undergraduate education, underrepresented minority

## Abstract

In 2020, the American Public Health Association declared structural racism a public health crisis acknowledging the long-lasting and harmful effects of prejudice, including relatively high rates of morbidity and mortality in many communities of color. Critical Race Theory (CRT) has become an essential lens to view and reconsider education’s role in perpetuating racial and ethnic discrimination. Debates over integrating CRT in higher education with the intent to acknowledge and address racial equality and justice are more present than ever, and the discussions held in public health classrooms are no different. We present a case study of CRT integration into the Bachelor of Arts in Public Health (BAPH) program at the University of Hawaiʻi at Mānoa. In line with Solorzano’s framework of CRT in education, initial goals of integrating CRT in instruction and advising included fostering discussions of race and racism, using a social justice framework to highlight opportunities to reduce health inequities, and validating the experiential knowledge of people of color. By engaging in active discussions with community leaders and participating in experiential learning throughout the program, students develop empathy and many underrepresented and marginalized students engage actively in their home communities. Specific examples of CRT integrated in the curriculum and examples of student projects that integrate a CRT lens are provided for educators and researchers.

## 1. Introduction

A bold statement by the American Public Health Association declared structural racism a public health crisis, worthy of immediate attention and research by public health practitioners and institutions (1). This 2020 policy was formed within the context of nationwide protests of racist law enforcement and criminal justice systems (2), an unprecedented presidential election mired in racist and xenophobic allegations (3, 4), and the heights of a pandemic which disproportionately affected people of color (5–9). These issues have been subsumed into an impassioned examination of Critical Race Theory (CRT) and its application.

Besides a robust body of scholarship examining American law and legal systems through a lens of historic and structural racism (10, 11), CRT has been used to reinterpret “colorblind” laws, policies, and systems that have paradoxically contributed to more discriminatory outcomes (12, 13). Fiery debates over integrating CRT in higher education with the intent to acknowledge and address racial equality and justice—particularly for Black, Indigenous, and People of Color (BIPOC) communities—are more present than ever (10, 14). Various opponents contend that shaping general education around the social construct of race and concepts of intersectionality creates a revisionist history hyper-focused on racial strife and unbalanced power structures (10, 15). In 17 states, laws restricting discussions of race and racism in primary and secondary school classrooms have been passed mainly to limit the discomfort of students (16). Others argue that the application of CRT is more important than ever in higher education as faculty seek to empower students with a more critical lens to examine the structures that continue to create social, health, and economic disparities across the country. Regardless, conversations on race continue to occur in academic spaces, the media, and everyday encounters.

The discussions held in medical and public health classrooms are no different (14, 17, 18). Since public health is grounded in the ethos of social justice and the collective stories of how people have survived and thrived, it is important to acknowledge those who have not thrived, often the disadvantaged and marginalized. Health inequalities remain rampant in this golden age of medical science (19). Racial and ethnic minority groups, throughout the US, are disproportionately more likely to suffer and die from a wide range of health conditions, including diabetes, hypertension, obesity, asthma, and heart disease, when compared to White counterparts. The life expectancy of non-Hispanic/Black Americans is 4 years lower than that of White Americans (20). The COVID-19 pandemic has exacerbated these disparities; racial and ethnic minority populations are disproportionately more likely to contract and die from the illness (6, 9). Public health pedagogy has integrated the social determinants of health (SDOH) model to highlight where, when, and among whom do inequalities arise.

The SDOH are the “conditions in the environments where people are born, live, learn, work, play, worship, and age.” (21) Communities of color are more likely to be born and live in unhealthy conditions. For example, there is a growing number of studies that confirm that unhealthy food outlets are more likely to be in Black and Native communities, while healthy food outlets are more likely to be available in predominantly white communities (22–24). But SDOH misses the larger historical context that continues to reinforce these inequitable conditions, thus requiring distillation through a framework grounded in socio-political realities, such as CRT (10, 14, 17, 25, 26).

Critical race theory provides students the tools to examine why these inequities are extant and perpetuated—and, subsequently, how they can address such inequities to improve community health. For example, CRT equips students to understand the historical policies and practices that have led to the conditions where Black and Native communities are more likely to live in communities with poor access to healthy foods and viable economic opportunities, quality schools, and safe neighborhoods. CRT allows us to understand how the US history of redlining and racially based mortgage loans have led to these inequitable conditions that have produced today’s health and socioeconomic disparities (27, 28).

The stories of the oppressed also serve as records of forced but invaluable contributions to the field of population health. For centuries, researchers have unethically experimented upon minoritized populations to discern the devastating effects of diseases—and yet these communities are woefully underrepresented in modern clinical trials of lifesaving pharmaceuticals (29, 30). CRT provides students with the capacity to question how these unethical breaches have occurred and why minorities are underrepresented in clinical research. Using the lens of CRT, students can investigate how research and science have been used to justify racist beliefs and policies, including justifying slavery, genocide, and colonization (31–33).

Baseless race-based myths regarding pain tolerance to disease transmission are still prevalent in medical education and among practicing clinicians (34, 35). Minority communities continue to be targeted and susceptible to misinformation and disinformation campaigns. Patients and providers are susceptible to myths perpetuating suboptimal population health (8). Only by confronting these uncomfortable truths about trenchant prejudice in healthcare access, delivery, and utilization can we start to build an institution worthy of training the next generation of public health practitioners.

Hawaiʻi is one of the most racially and ethnically diverse states in the US. In 2019, 24.2% of Hawaiʻi’s population reported being multiracial compared to only 2.8% of the US population. Hawaiʻi is the ancestral home to the Indigenous population of Native Hawaiians or Kānaka Maoli, who comprise slightly over 10% of the population, including other Pacific Islanders who have migrated to Hawaiʻi (e.g., Samoans, Tongans, Micronesians). Asians, such as Japanese, Chinese, Korean, Filipinos, compose approximately 38% of the population. Hawaiʻi’s Hispanic and Black or African American populations are proportionally smaller than the continental United States (36). Although Hawaiʻi is often portrayed as one of the healthiest states in the United States, pervasive health disparities exist across these racial and ethnic groups. Factors such as colonization, structural racism, and assimilation contribute to inequitable access to housing, healthcare, education, and occupation. For example, Native Hawaiians and other Pacific Islanders have one of the highest rates of diabetes and hypertension compared with other major racial groups (37). Native Hawaiians have the shortest life expectancy—only 62 years—in their own homeland (38).

Training the next generation of Hawaiʻi public health leaders to address these social and health disparities will require a dynamic curriculum grounded in CRT’s social justice principles. We need to cultivate an increasingly diverse student body that reflects the communities who are most affected by these disparities. While the SDOH model does much to support curricula, it misses elements to identify rooted inequities that determine health. The addition of CRT tools is essential in any undergraduate public health program, even though the discipline claims to see and address inequity. Here we present a perspective of such a merger between philosophies in the Bachelor of Arts in Public Health (BAPH) program at the Office of Public Health Studies (OPHS) at the University of Hawaiʻi at Mānoa (UHM).

## 2. Program background

The University of Hawaiʻi at Mānoa was established in 1907 as a land-grant university ‌on the island of Oʻahu. A part of UHM’s strategic plan is to become a Hawaiian place of learning. The university is also classified as an Asian-American, Native-American, and Pacific Islander-Serving Institution (AANAPISI) (39). Among the undergraduate student body, about 33% are Asian and 18% are Native Hawaiian and Pacific Islander (40).

Office of Public Health Studies, under the Thompson School of Social Work and Public Health, offers specializations in epidemiology, health policy and management, Native Hawaiian and Indigenous health, and social and behavioral health sciences (41). Degrees offered include the Bachelor of Arts (BA), Master of Public Health (MPH), Master of Science (MS), and Doctor of Philosophy (PhD). Proactive academic advising serves to promote the enrollment and retention of diverse students, while targeted recruitment and outreach efforts to underrepresented communities have been fruitful.

The BAPH program started in 2014, in response to a nationwide expansion in demand for undergraduate education (42–44), with inclusion in mind and using culturally relevant curriculum, aids, models, and examples taught by diverse faculty and staff. This curriculum, rooted in SDOH, has always included diverse perspectives, valued student lived experiences, and featured a range of locally relevant cultural examples. Beginning in the Fall 2020 semester, the curriculum expanded and scaled its previously fragmented application of CRT tools more intentionally and purposefully to respond to the need for anti-racist education. Since its inception, enrollment has grown from 35 students in 2015 to over 150 students in 2022. Our undergraduate student semester hours have been increasing since 2018, and enrollment in the public health minor has also grown since its launch in Fall 2018 to a consistent cohort size of roughly 30 students per academic year, demonstrating our department’s interdisciplinary reach. Approximately 50% of the BAPH student body is Native Hawaiian, other Pacific Islander, Indigenous, and/or Filipino ancestries, and 65% are Hawaiʻi residents (45).

## 3. Critical race theory in public health education

Solórzano provides five themes that guide CRT in education: (a) centering discussions of race and racism, (b) recognizing the dominant ideologies and challenging the claims toward a more just educational system, (c) using a social justice framework to end oppression, (d) validating the experiential knowledge of people of color, and (e) utilizing the interdisciplinary nature of CRT to frame racism within historical and contemporary contexts of people of color (46). As listed in Table 1, numerous examples in the BAPH program incorporate CRT within the courses and in advising and support. By centering race and racism in discussions throughout the BAPH curriculum, students begin to think critically about the systems of oppression that disproportionately impact people of color.

**Table 1:** Applications of critical race theory in public health classroom settings.

Course	Core curricular content	CRT application and select examples used
PH 201: Introduction to public health	Ethical violations in research and practice	Oral contraceptive experimentation among Puerto Rican women (47) Kalaupapa and mistreatment of Hansen’s Disease patients in Hawai‘i (48) Tuskegee Syphilis Study (49, 50)
PH 201: Introduction to public health	Built environment	Inequities in the built environment (51) Discussion of red-lining and both short and long-term consequences among BIPOC communities (28, 52) Discussion of built environment interventions in low-income neighborhoods (53)
PH 202: Public health issues in Hawai‘i	Food insecurity	Environmental impact of food importation and dependence (54) Sustainability of historical and cultural practices (e.g., ahupua‘a system) (55) History of farming and community connections (56)
PH 202: Public health issues in Hawai‘i	History of public health in Hawai‘i	Hawaiian monarchy’s role in developing health department prior to US occupation (57) Hawaiian monarchy investment and development of preeminent Queen’s Medical Center (57)
PH 202: Public health issues in Hawai‘i	Plantation history	Unionization of plantation workers (i.e., primarily immigrant communities) (58) Health disparities in plantation communities (59)
PH 202: Public health issues in Hawai‘i	Epidemiology in Hawai‘i	Disproportionate impacts of COVID-19 infection and mortality on Native Hawaiians and Pacific Islanders (60)
PH 203: Introduction to global health	Global health history, geography, and colonization	Use of geographical maps depicting Indigenous communities pre-colonization (61) Student viewing and reflection of “High on the Hog” documentary (62)
PH 203: Introduction to global health	Vaccination distribution and vaccine hesitancy	Genocide in present-day Namibia, early 1900s by German officials (63) Drug companies conducted research trials without consent, e.g., Pfizer in Nigeria (64) HIV treatment research in southern Africa (65–69)
PH 203: Introduction to global health	Women and girls’ education	Cultural barriers to education for women (70, 71) Barriers to education associated with limited access to feminine hygiene products, sanitation, and bathrooms (72–74)
PH 203: Introduction to global health	Maternal health	Systemic barriers to accessing healthcare among women (75) Lack of prenatal care and limited access to skilled birth attendants among people of color (76) Cultural and religious barriers to contraception (77) Impacts of obstetric fistula in Namibia (Africa) (78) Labor and delivery challenges in Manila (Philippines) (79), both highlighting firsthand, lived experience accounts
PH 203: Introduction to global health	Conflict and refugees	Bias and racism in media coverage of war and conflict (e.g., Ukrainian refugee coverage compared to Tigray and Yemen humanitarian crises) (80–82)

The introductory core series at the BAPH program provides students a broad-based foundation of public health knowledge and skills (83). These courses also offer an opportunity to introduce fundamental concepts that allow students to build their character and discover their whole selves, empowering them to engage in anti-racist critical thinking in public health through community work and advocacy.

For example, in PH 201: Introduction to Public Health course, CRT frameworks are utilized while introducing topics such as health disparities, ethical research, climate change, and the built environment. The course highlights the impact of colonization on the health of Native Hawaiian people through facilitated discussions on health disparities and Native Hawaiian health. Examples of ethical violations center on the marginalization of BIPOC communities and women. The lived experiences of Pacific Island communities allow students to connect the injustices these populations face. The historical experiences of Black communities—where U.S. redlining policies and lack of community investment continue to prevent economic mobility—are discussed within conversations about inequities found in Hawaiʻi.

PH 202 is a course focused on Public Health Issues in Hawai‘i. With UHM committed as an Indigenous-serving institution (39), it is important to have a course that ties together the lived experiences and historical context of the Native Hawaiian people as it relates to public health. The course covers key historical events of Hawai‘i, including pre-western contact, impact of land tenureship as a result of external forces such as the arrival of missionaries, plantation culture, and the illegal overthrow of the Hawaiian kingdom, and how these events impact Indigenous peoples and their land (58). In more recent history, increased attention is paid to inequities among Pacific Island communities of migrants from Compact of Free Association (COFA) countries, who participate in the labor force and have waning access to federal healthcare and assistance programs yet face harsh discrimination from other groups (84, 85). Guest speakers from these communities are intentionally invited to share their stories, research, and expertise that centers their experiential knowledge.

The course weaves the topic of colonial impact throughout PH 203: Introduction to Global Health Issues. By providing the historical and contemporary impacts of colonization on BIPOC communities, students learn to identify and associate systemic oppressions as the direct consequences of colonization and historical trauma. These consequences include dietary shifts from traditional food systems contributing to rises in non-communicable diseases; impacts of climate change disproportionately impacting people of color, especially island communities; internalized racism as illustrated by shifts towards western beauty standards driven by colonization; and losing cultural practices that once maintained community wellness.

Throughout the semester, students engage in active discussions and experiential learning activities such as water-carrying exercises to illustrate negative consequences of water access on BIPOC communities and the disproportionate burden of water-carrying on women and girls. Women and girls are overwhelmingly responsible for unpaid domestic work including water collection. In fact, globally, women spend a large proportion of their time and energy waiting for, securing, and transporting water (86). Students also develop written policy and role-play policy-making discussions through a modified Model United Nations process. These activities help empower students to identify examples of systemic racism and associated health disparities, then locate or develop policy proposals to dismantle existing systems. The course also shares documentaries portraying lived experiences of BIPOC communities to spur continual student reflection. Students also learn about the impacts of climate change that led to migration of climate change refugees.

With a strong foundation in the introductory core, students are well-positioned and prepared for the applied learning experience (APLE). Students in the BAPH program complete a three-course capstone series where they apply classroom knowledge and associated skills to real-world applications in the public health field (87). The first course of the APLE series allows students to reflect on their own experiences and complete a literature review in an area of public health of their choice. The instructors introduce key research skills using a social justice framework, with the intent of helping students identify selection and publication biases in literature, and the dangers of misclassifying underrepresented minorities in academic studies. In the second course, students complete a 100-h field experience with a community or faculty mentor, then in the third and final course, reflect on and finalize a written paper and academic poster. All three courses are centered on a public health issue of the student’s selection (87).

The power of choice and supporting theories of student self-authorship empower students to think critically about different communities’ issues and explore public health solutions to address them. Baxter Magolda defines self-authorship as the ability to control and orient one’s beliefs and identity through concurrent membership in multiple communities like families, workforces, and student bodies (88). Encouraging underrepresented and marginalized students to engage actively in their home communities is a validating experience that raises their consciousness of the cultural capitals they bring as leaders. Table 2 highlights various student APLE projects integrating a CRT lens.

**Table 2:** Examples of bachelors of public health student-developed capstone projects, 2021–2022: applying themes of critical race theory.

Public health student-developed capstone project titles	Solórzano framework (98) of critical race theory in education				
	Centrality and intersectionality of race and racism	Challenge to dominant ideology	Commitment to social justice	Centrality of experiential knowledge	Interdisciplinary perspective
Reducing racial disparities in maternal mortality among black women in the United States	**X**		**X**	**X**	
Improving access to quality care among Filipino women in the Philippines to reduce maternal mortality			**X**	**X**	
Addressing type II Diabetes among native Americans by promoting a cultural diet	**X**	**X**	**X**	**X**	**X**
Preventing type II Diabetes among native Hawaiians by increasing access to produce in Hawaiʻi	**X**			**X**	**X**
Increasing breast cancer screening through culturally appropriate programs among native Hawaiian women in Hawai‘i	**X**	**X**	**X**		**X**
Raising COVID-19 vaccination rates among pacific islander populations within Hawai‘i through outreach and education programs	**X**				**X**
Decreasing COVID-19 infection among native Hawaiians and Pacific islanders in rural communities by promoting use of testing in school settings	**X**			**X**	
Preventing obesity among native Hawaiian adults in Hawai‘i through the promotion of a cultural diet	**X**	**X**	**X**		**X**
preventing hypertension among Filipinos in Hawai‘i by increasing access to healthy foods	**X**			**X**	**X**
Decreasing chlamydia in black, indigenous, people of color teens in Hawai‘i through early screening and treatment	**X**		**X**	**X**	**X**
Promoting early detection of breast cancer among Filipino adult women in Hawai‘i through screenings and health literacy	**X**			**X**	**X**

## 4. Critical race theory in advising and student support

Many factors can impact a student’s sense of belonging and success in higher education. Advisors play a crucial role for students, especially those of underrepresented backgrounds, by providing a relationship and environment to help affirm, support, and advocate for students’ needs (89). Advisors are gatekeepers of information and opportunities that can provide equitable experiences for students of marginalized backgrounds. The BAPH program focuses on recruiting and supporting students from underrepresented populations in higher education, including Native Hawaiian, Pacific Islander, Filipino, and other Indigenous backgrounds who are more likely to be first-generation college students. The advisor and faculty members who support the BAPH program are from underrepresented backgrounds themselves (e.g., first-generation college graduates, and ethnic or religious minorities) and possess a deep understanding of the structural barriers these populations face in accessing and pursuing higher education. These faculty members are able to contribute their own lived experiences and, by doing so, are better equipped to support students in finding their own voice and language to share their stories. By centering students’ lived experiences, a CRT approach in advising encourages dialogue that highlights students’ strengths rooted in their cultural capitals (90).

Advising public health students with a CRT approach includes honoring students’ narratives and histories while providing a space to discuss altruistic, prosocial sociocultural goals. Conversations in the classroom provide rich opportunities during advising sessions to reflect on key public health topics. Within a CRT approach, advisors affirm students’ experiences and any injustices suffered while focusing on students’ strengths and commitments to serving the community and contributing to the greater good. The overall goal of advising is to ensure students’ academic, emotional, familial, and economic needs are addressed and supported through trust built on advisor-student rapport.

## 5. Discussion

Centering the lived experiences of the oppressed and marginalized is essential to address the public health issues that exist due to injustices and inequities throughout history and those in the present day. The levels of racism that contribute to the race-associated differences in health outcomes is a recognized practice in health equity work (91). Public health research and practice continue integrating CRT to develop programs and interventions (92, 93). Undergraduate public health education is well-positioned to integrate the frameworks of CRT into the curriculum. In turn, programs will develop future public health leaders equipped to recognize the impact of dominant racist ideologies and powers that lead to systemic racism and health disparities.

Curriculum reform through a CRT lens can support the development of students’ global identities and skills to communicate, respect, and understand others (94). A special issue of the *Australian Journal of Indigenous Education* highlights higher education’s role in building an Indigenous health workforce (95), including addressing curriculum gaps in Indigenous public health (96). Our case study contributes to the growing literature on social justice and antiracism pedagogy in public health education (97).

The following are recommendations for integrating CRT in a public health curriculum. Normalizing conversations about race is essential in public health work. Beyond competencies needed to fulfill accreditation, faculty should include anti-racism training as a learning outcome in various courses. Providing students with these experiences in introductory courses can help to prepare them for deeper, more critical applications of the theory in later courses and in practice. Furthermore, the curriculum should be intentional about the examples in lectures and assignments. Faculty should also invite guest speakers to share voices and stories from underrepresented communities. Holding our faculty, staff, and students to an anti-racist ideal will normalize the practice of addressing racism as a fundamental public health issue.

## 6. Conclusion

Students study public health at the undergraduate level to become public health professionals but also as a complement to other health professional pathways. Many students will study public health and then engage in direct community service; others will continue on in allied health fields, such as nursing, medicine, and pharmacy. In any event, applications of critical race and related educational theories are essential to surround students with the indispensable context of historical and current systemic barriers to the principles of community health, equity, and social justice. Integrating CRT frameworks into undergraduate public health education creates a more anti-racist, socially aware, and empowered public health workforce vital to addressing ongoing community challenges and promoting health equity.

## Data availability statement

The original contributions presented in the study are included in the article/supplementary material, further inquiries can be directed to the corresponding author.

## Author contributions

MT-K, UP, and DN-H contributed conception and design of the project. UP and MT-K wrote the first draft of the manuscript. MT-K, UP, JC-D, LK, MA, and DN-H wrote sections of the manuscript. All authors contributed to the manuscript revision, read, and approved the submitted version.

## Conflict of interest

The authors declare that the research was conducted in the absence of any commercial or financial relationships that could be construed as a potential conflict of interest.

## Publisher’s note

All claims expressed in this article are solely those of the authors and do not necessarily represent those of their affiliated organizations, or those of the publisher, the editors and the reviewers. Any product that may be evaluated in this article, or claim that may be made by its manufacturer, is not guaranteed or endorsed by the publisher.

## References

[1] American Public Health Association. Structural Racism is a Public Health Crisis: Impact on the Black Community [internet]. Washington, DC: American Public Health Association (2020).

[2] American Public Health Association. Violence is a Public Health Issue: Public Health is Essential to Understanding and Treating Violence in the U.S. [internet]. Washington, D.C.: American Public Health Association (2018).

[3] Anderson-Nathe B Gharabaghi K. Trending rightward: nationalism, xenophobia, and the 2016 politics of fear. Child Youth Serv. (2017) 38:1–3. doi: 10.1080/0145935X.2017.1277125

[4] Darling-Hammond L. Teaching for social justice: resources, relationships, and anti-racist practice. Multicult Perspect. (2017) 19:133–8. doi: 10.1080/15210960.2017.1335039

[5] Büyüm AM Kenney C Koris A Mkumba L Raveendran Y. Decolonising global health: if not now, when? BMJ Glob Health. (2020) 5:e003394. doi: 10.1136/bmjgh-2020-003394, PMID: 32759186 PMC7409954

[6] Centers for Disease Control and Prevention. Introduction to COVID-19 racial and ethnic health disparities [internet]. Ctr Dis Control Prev. (2020)

[7] Kendi IX. We still Don’t know who the Coronavirus’s victims were. The Atlantic [Internet]. (2021)

[8] Quint JJ Van Dyke ME Maeda H Worthington JK Dela Cruz MR Kaholokula JK. Disaggregating data to measure racial disparities in COVID-19 outcomes and guide community response—Hawaii, March 1, 2020–February 28, 2021. MMWR Morb Mortal Wkly Rep. (2021) 70:1267–73. doi: 10.15585/mmwr.mm7037a1, PMID: 34529634 PMC8445382

[9] The Atlantic Monthly Group. About the racial data tracker [internet]. The COVID Tracking Project. (2022)

[10] Delgado R Stefancic J Harris A. Critical Race Theory: An Introduction [internet]. Third. New York, NY: New York University Press (2017).

[11] Crenshaw KW. Twenty years of critical race theory: looking Back to move forward. Conn Law Rev. (2011) 43:103.

[12] Johnson TR. How conservatives turned the ‘color-blind constitution’ against racial Progress. The Atlantic [Internet]. (2019)

[13] Annamma SA Jackson DD Morrison D. Conceptualizing color-evasiveness: using dis/ability critical race theory to expand a color-blind racial ideology in education and society. Race Ethn Educ. (2017) 20:147–62. doi: 10.1080/13613324.2016.1248837

[14] Lightfoot AF Efird CR Redding EM. Developing an antiracist Lens: using photography to facilitate public health critical race praxis in a foundational MPH course. Pedagogy Health Promot. (2021) 7:317–26. doi: 10.1177/23733799211045712

[15] Neville HA Gallardo ME Sue DW eds. Introduction: Has the United States Really Moved Beyond Race? In: The Myth of Racial Color Blindness: Manifestations, Dynamics, and Impact [internet]. Washington, DC: American Psychological Association (2016). 3–21.

[16] Pendharkar E. A school openly discusses race in a state that bans it. Education Week [Internet]. (2022)

[17] Zaidi Z Young M Balmer DF Park YS. Endarkening the Epistemé: critical race theory and medical education scholarship. Acad Med. (2021) 96:Si–v. doi: 10.1097/ACM.0000000000004373 34432718

[18] Fletcher FE Jiang W Best AL. Antiracist praxis in public health: a call for ethical reflections. Hast Cent Rep. (2021) 51:6–9. doi: 10.1002/hast.1240, PMID: 33840102 PMC9009418

[19] Walensky RP. Director’s Commentary [internet]. Bethesda, MD: Centers for Disease Control and Prevention (2021).

[20] Centers for Disease Control and Prevention. Racism and Health [Internet]. Bethesda, MD: Centers for Disease Control and Prevention (2021).

[21] Office of Disease Prevention and Health Promotion. Social Determinants of Health - Healthy People 2030 [Internet]. (n.d.). [cited 2022 Jan 14]. Available from: https://health.gov/healthypeople/objectives-and-data/social-determinants-health

[22] Cooksey Stowers K Jiang Q Atoloye AT Lucan S Gans K. Racial differences in perceived food swamp and Food Desert exposure and disparities in self-reported dietary habits. Int J Environ Res Public Health. (2020) 17:7143. doi: 10.3390/ijerph17197143, PMID: 33003573 PMC7579470

[23] Fleischhacker SE Evenson KR Rodriguez DA Ammerman AS. A systematic review of fast food access studies. Obes Rev. (2011) 12:e460–71. doi: 10.1111/j.1467-789X.2010.00715.x 20149118

[24] Lee S Oshiro M Hsu L Buchthal OV Sentell T. Neighborhoods and health in Hawai‘i: considering food accessibility and affordability. Hawaii J Med Public Health. (2012) 71:232–7.

[25] Tsai J Lindo E Bridges K. Seeing the window, finding the spider: applying critical race theory to medical education to make up where biomedical models and social determinants of health curricula fall short. Front Public Health [Internet]. (2021) 9. doi: 10.3389/fpubh.2021.653643/full, PMID: 34327185 PMC8313803

[26] Carroll SR Suina M Jäger MB Black J Cornell S Gonzales AA. Reclaiming indigenous health in the US: moving beyond the social determinants of health. Int J Environ Res Public Health. (2022) 19:7495. doi: 10.3390/ijerph19127495, PMID: 35742745 PMC9223447

[27] Yearby R. Racial disparities in health status and access to healthcare: the continuation of inequality in the United States due to structural racism. Am J Econ Sociol. (2018) 77:1113–52. doi: 10.1111/ajes.12230

[28] Nardone A Chiang J Corburn J. Historic redlining and urban health today in U.S. Cities Environ Justice. (2020) 13:109–19. doi: 10.1089/env.2020.0011

[29] Lefor AT. Scientific misconduct and unethical human experimentation: historic parallels and moral implications. Nutrition. (2005) 21:878–82. doi: 10.1016/j.nut.2004.10.011, PMID: 15975498

[30] Hussain-Gambles M. Ethnic minority under-representation in clinical trials. Whose responsibility is it anyway? J Health Organ Manag. (2003) 17:138–43. doi: 10.1108/14777260310476177 12916177

[31] Harvard Library. Scientific racism [internet]. Harvard Library (2022). Available at: https://library.harvard.edu/confronting-anti-black-racism/scientific-racism

[32] Weigmann K. In the name of science. EMBO Rep. (2001) 2:871–5. doi: 10.1093/embo-reports/kve217, PMID: 11600445 PMC1084095

[33] Brave Heart MYH Chase J Elkins J Altschul DB. Historical trauma among indigenous peoples of the Americas: concepts, research, and clinical considerations. J Psychoactive Drugs. (2011) 43:282–90. doi: 10.1080/02791072.2011.628913, PMID: 22400458

[34] Chapman EN Kaatz A Carnes M. Physicians and implicit Bias: how doctors may unwittingly perpetuate health care disparities. J Gen Intern Med. (2013) 28:1504–10. doi: 10.1007/s11606-013-2441-1, PMID: 23576243 PMC3797360

[35] Hall WJ Chapman MV Lee KM Merino YM Thomas TW Payne BK. Implicit racial/ethnic Bias among health care professionals and its influence on health care outcomes: a systematic review. Am J Public Health. (2015) 105:e60–76. doi: 10.2105/AJPH.2015.302903, PMID: 26469668 PMC4638275

[36] Research Economic Analysis Division. Hawaii Population Characteristics 2019 [Internet]. (2020). Available at: https://census.hawaii.gov/wp-content/uploads/2020/06/Hawaii-Population-Characteristics-2019.pdf

[37] Look MA Soong S Kaholokula JK. Assessment and Priorities for Health and Well-being in Native Hawaiians and Pacific Islanders [internet]. Honolulu, HI, US: Department of Native Hawaiian Health, John A. Burns School of Medicine, University of Hawaiʻi (2020).

[38] Wu Y Braun K Onaka AT Horiuchi BY Tottori CJ Wilkens L. Life expectancies in Hawai‘i: a multi-ethnic analysis of 2010 life tables. Hawaii J Med Public Health. (2017) 76:9–14. PMID: 28090398 PMC5226016

[39] University of Hawaiʻi at Mānoa. About UH Mānoa [Internet]. (2023). Available from: https://manoa.hawaii.edu/about/

[40] University of Hawaiʻi at Mānoa, Office of Student Equity, Excellence and Diversity. Mānoa’s Racial and Ethnic Diversity Profile [internet]. Honolulu, HI: University of Hawaiʻi at Mānoa (2016). 7 p.

[41] Office of Public Health Studies. About [internet]. University of Hawaiʻi at Mānoa. (2022). Available at: http://manoa.hawaii.edu/publichealth/about

[42] Leider JP Castrucci BC Plepys CM Blakely C Burke E Sprague JB. On academics: characterizing the growth of the undergraduate public health major: U.S., 1992–2012. Public Health Rep. (2015) 130:104–13. doi: 10.1177/003335491513000114, PMID: 25552763 PMC4245293

[43] Resnick B Leider JP Riegelman R. The landscape of US undergraduate public health education. Public Health Rep. (2018) 133:619–28. doi: 10.1177/0033354918784911, PMID: 30084738 PMC6134569

[44] Riegelman RK. 2022 Update On Undergraduate Public Health [internet]. Burlington, MA: Jones & Bartlett Learning (2022).

[45] Mānoa Institutional Research Office. University of Hawaiʻi at Mānoa enrollment trend report [internet]. (2022). Available at: https://manoa.hawaii.edu/miro

[46] Solorzano DG. Critical race theory, race and gender microaggressions, and the experience of Chicana and Chicano scholars. Int J Qual Stud Educ. (1998) 11:121–36. doi: 10.1080/095183998236926

[47] Vargas T. Guinea pigs or pioneers? How Puerto Rican women were used to test the birth control pill. *Washington Post [Internet]*. (2017); Available at: https://www.washingtonpost.com/news/retropolis/wp/2017/05/09/guinea-pigs-or-pioneers-how-puerto-rican-women-were-used-to-test-the-birth-control-pill/

[48] Wong A. People with leprosy were exiled there—Should it be a tourist destination? The Atlantic [internet]. (2015); Available at: https://www.theatlantic.com/health/archive/2015/05/when-the-last-patient-dies/394163/

[49] Centers for Disease Control and Prevention. Tuskegee Study [Internet] (2021). Available at: https://www.cdc.gov/tuskegee/timeline.htm

[50] Lombardo PA Dorr GM. Eugenics, medical education, and the public health service: another perspective on the Tuskegee syphilis experiment. Bull Hist Med. (2006) 80:291–316. doi: 10.1353/bhm.2006.0066, PMID: 16809865

[51] Hutch DJ Bouye KE Skillen E Lee C Whitehead L Rashid JR. Potential strategies to eliminate built environment disparities for disadvantaged and vulnerable communities. Am J Public Health. (2011) 101:587–95. doi: 10.2105/AJPH.2009.173872, PMID: 21389288 PMC3052324

[52] Jackson C. What is redlining? *The New York Times [Internet]* (2021); Available at: https://www.nytimes.com/2021/08/17/realestate/what-is-redlining.html

[53] Branas CC South E Kondo MC Hohl BC Bourgois P Wiebe DJ. Citywide cluster randomized trial to restore blighted vacant land and its effects on violence, crime, and fear. Proc Natl Acad Sci. (2018) 115:2946–51. doi: 10.1073/pnas.1718503115, PMID: 29483246 PMC5866574

[54] Ritchie H Roser M. Environmental impacts of food production. *Our World Data [Internet]*. (2020); Available at: https://ourworldindata.org/environmental-impacts-of-food

[55] Gon S Winter K. A Hawaiian renaissance that could save the world. American Scientist [Internet]. (2019) 107:232. doi: 10.1511/2019.107.4.232

[56] University of Northern Iowa. Farming for public health | evidence-based farming! [internet]. (2022). Available at: https://farmingforpublichealth.org/

[57] Anonymous. History of Hawaii’s public health [internet]. (2015). Available at: https://www.youtube.com/watch?v=rJEIqGMaasw

[58] McDermott JF Andrade NN eds. People and Cultures of Hawaiʻi: The Evolution of Culture and Ethnicity [internet]. Honolulu: University of Hawaiʻi Press (2011). 347 p.

[59] Wikipedia Authors. Picture Bride (film). In: Wikipedia [internet]. (2022). Available at: https://en.wikipedia.org/w/index.php?title=Picture_Bride_(film)&oldid=1096673329

[60] Hofschneider A. Pacific islanders, Filipinos have highest COVID-19 rates in Hawaii. *Honolulu Civil Beat [Internet]*. (2020); Available at: https://www.civilbeat.org/2020/06/pacific-islanders-filipinos-have-highest-covid-19-rates-in-hawaii/

[61] Fields S. Decolonizing the map: Creating the indigenous mapping… [internet]. PBS Education. (2022). Available at: https://www.pbs.org/education/blog/decolonizing-the-map-creating-the-indigenous-mapping-collective

[62] Anonymous.High on the hog: how African American cuisine transformed America [internet] (2021). Available at: https://www.netflix.com/title/81034518

[63] United States Holocaust Memorial Museum. Herero and Nama Genocide [Internet] (2022). Available at: https://www.ushmm.org/collections/bibliography/herero-and-nama-genocide

[64] Lenzer J. Secret report surfaces showing that Pfizer was at fault in Nigerian drug tests. BMJ. (2006) 332:1233. doi: 10.1136/bmj.332.7552.1233-a, PMID: 16735322 PMC1471980

[65] McNeil DG. A failed trial in Africa raises questions about how to test H.I.V. *Drugs The New York Times [Internet]* (2015); Available at: https://www.nytimes.com/2015/02/05/health/failed-trial-in-africa-raises-questions-about-how-to-test-hiv-drugs.html

[66] Clark K. Clinical trials in developing countries: the new “Tuskegee.”. WR J Arts Sci Writ Program [Internet]. (2011) 4

[67] Annas GJ Grodin MA. Human rights and maternal-fetal HIV transmission prevention trials in Africa. Am J Public Health. (1998) 88:560–3. doi: 10.2105/AJPH.88.4.560, PMID: 9550993 PMC1508440

[68] Brewster D. Science and ethics of human immunodeficiency virus/acquired immunodeficiency syndrome controversies in Africa. J Paediatr Child Health. (2011) 47:646–55. doi: 10.1111/j.1440-1754.2011.02179.x, PMID: 21951451

[69] Cunningham B. Partially treated: AIDS, inequality and ethics: The controversy over the short course AZT trials [Internet] [Dissertation]. [Ann Arbor, MI, US]: University of Michigan. (2006). Available from: https://www.proquest.com/dissertations-theses/partially-treated-aids-inequality-ethics/docview/305278829/se-2

[70] Yousafzai M. I Am Malala. Boston, MA: Little, Brown (2013). 352 p.

[71] Singmaster H. The legacy of Michelle Obama and the let girls learn initiative. *Education Week [Internet]* (2017); Available at: https://www.edweek.org/leadership/opinion-the-legacy-of-michelle-obama-and-the-let-girls-learn-initiative/2017/01

[72] Sanchez E Rodriguez L. Period poverty: everything you need to know [internet]. *Global Citizen* (2019). Available at: https://www.globalcitizen.org/en/content/period-poverty-everything-you-need-to-know/

[73] UNESCO. Puberty Education and Menstrual Hygiene Management [internet]. Paris, FR: UNESCO (2014). 58 p.

[74] Lusk-Stover O Rop R Tinsley E Rabie TS. Globally, periods are causing girls to be absent from school [internet]. (2016). Available at: https://blogs.worldbank.org/education/globally-periods-are-causing-girls-be-absent-school

[75] Alexander LL LaRosa JH Bader H Garfield S. New Dimensions in Women’s Health Jones & Bartlett Learning (2020). 504 p.

[76] Artiga S Orgera K Pham O Corallo B. Growing data underscore that communities of color are being harder hit by COVID-19 [internet]. *Kaiser Family Foundation*; (2020). (Disparities Policy Brief). Available at: https://www.kff.org/coronavirus-policy-watch/growing-data-underscore-communities-color-harder-hit-covid-19/

[77] Srikanthan A Reid RL. Religious and cultural influences on contraception. J Obstet Gynaecol Can. (2008) 30:129–37. doi: 10.1016/S1701-2163(16)32736-0 18254994

[78] Anonymous. Shout Gladi Gladi [Internet]. (2015). Available at: https://www.shoutgladigladi.com/

[79] Motherland [Internet]. CineDiaz. (2017) [cited 2023 Aug 13]. Available from: https://www.imdb.com/title/tt6333078/

[80] Ryder M. The media’s racial bias is also happening off screen. Fortune [internet]. (2022); Available at: https://fortune.com/2022/03/10/racial-bias-ukraine-war-reporting-media-diversity-trust-marcus-ryder/

[81] Ali L. In Ukraine reporting, Western press reveals grim bias toward “people like us.” *Los Angeles Times [Internet]* (2022); Available at: https://www.latimes.com/entertainment-arts/tv/story/2022-03-02/ukraine-russia-war-racism-media-middle-east

[82] Mba Abogo CA. Racism in Western reporting of the Ukraine war. How should we respond? *New African Magazine [Internet]* (2022); Available at: https://newafricanmagazine.com/27904/

[83] Nelson-Hurwitz DC Tagorda M Kehl L Buchthal OV Braun KL. Developing an undergraduate public health introductory Core course series. Front Public Health. (2018) 6:155. doi: 10.3389/fpubh.2018.00155, PMID: 29892596 PMC5985697

[84] Blair C. Micronesian immigration an “important civil rights issue” facing Hawaii—Honolulu civil beat. (2015); Available at: https://www.civilbeat.org/2015/08/micronesian-immigration-an-important-civil-rights-issue-facing-hawaii/

[85] Hofschneider A. Report: battling discrimination against Micronesians requires policy changes. Honolulu Civil Beat [Internet]. (2019)

[86] Adams EA Adams YJ Koki C. Water, sanitation, and hygiene (WASH) insecurity will exacerbate the toll of COVID-19 on women and girls in low-income countries. Sustain Sci Pract Policy. (2021) 17:85–9. doi: 10.1080/15487733.2021.1875682

[87] Nelson-Hurwitz DC Tagorda M. Developing an undergraduate applied learning experience. Front Public Health [Internet]. (2015). doi: 10.3389/fpubh.2015.00002/abstract PMC4327175 25741503

[88] Baxter Magolda MB. Self-authorship. New Dir High Educ. (2014) 2014:25–33. doi: 10.1002/he.20092

[89] Lee JA. Affirmation, support, and advocacy: critical race theory and academic advising. NACADA J. (2018) 38:77–87. doi: 10.12930/NACADA-17-028

[90] Puroway A. Critical advising: a Freirian-inspired approach. NACADA J. (2016) 36:4–10. doi: 10.12930/NACADA-15-015

[91] Camara PJ. Levels of racism: a theoretic framework and a gardener’s tale. Am J Public Health. (2000) 90:1212–5. doi: 10.2105/AJPH.90.8.1212 10936998 PMC1446334

[92] Petteway RJ González LA. Engaging public health critical race praxis in local social determinants of Health Research: the youth health equity and action research training program in Portland, OR—yHEARTPDX. Int J Environ Res Public Health. (2022) 19:8187. doi: 10.3390/ijerph19138187, PMID: 35805851 PMC9266579

[93] Amani B Cabral A Sharif MZ Huỳnh J Skrine Jeffers K Baptista SA. Integrated methods for applying critical race theory to qualitative COVID-19 equity research. Ethn Dis. (2022) 32:243–56. doi: 10.18865/ed.32.3.243, PMID: 35909643 PMC9311305

[94] Azzahrawi R. A Look at the approaches to multicultural and anti-racist education through the lenses of critical race theory: the reported benefits and failures. J Adv Res Educ. (2023) 2:1–9. doi: 10.56397/JARE.2023.01.01

[95] Bond C Kajlich H. Introduction to the special issue: critical conversations on higher education as an enabler to building an indigenous health workforce. Aust J Indig Educ. (2020) 49:108–9. doi: 10.1017/jie.2020.12

[96] Lee N. Addressing the knowledge gap of indigenous public health: reflections from an indigenous public health graduate. Aust J Indig Educ. (2020) 49:110–8. doi: 10.1017/jie.2020.15

[97] Bentley KM Fortune D Rooks R Walter G. Antiracism and the pursuit of social justice. Pedagogy Health Promot. (2021) 7:296–8. doi: 10.1177/23733799211054402

